# Illness experience and coping strategies of young adults with inflammatory bowel disease: a qualitative study

**DOI:** 10.3389/fped.2026.1754064

**Published:** 2026-02-24

**Authors:** Jiaqi Zhang, Shurong Ren, Wenqin Ding, Jiefeng Yang

**Affiliations:** Department of Gastroenterology, The First Affiliated Hospital with Nanjing Medical University, Nanjing, Jiangsu, China

**Keywords:** patient experience, coping strategies, young adults, inflammatory bowel disease, Crohn's disease, ulcerative colitis, qualitative research

## Abstract

**Objective:**

To explore the illness experience and coping strategies of young adults with inflammatory bowel disease (IBD) to inform patient management.

**Methods:**

20 IBD patients aged 18–29 presenting for outpatient or inpatient treatment at the Gastroenterology Department of a tertiary hospital in Jiangsu Province between September 2023 and January 2024 were identified by purposive and snowball sampling and enrolled. A phenomenological approach was taken, semi-structured interviews conducted and interview data analyzed by Colaizzi's method.

**Results:**

Three themes were identified: (1) complex negative emotions arising from disrupted daily life, restricted self-development, uncertainty and anxiety about the future and conflicts between the desire for independence vs. the reality of dependence, (2) coping strategies that ranged from active coping to passive avoidance and (3) a multi-level need for external support from family, professionals and a wider social network.

**Conclusion:**

Young adults with IBD experience complex negative emotional responses. Active coping strategies may fostered personal development that transcended their pre-illness sense of self in young adults with IBD. The need for multi-level external support was clearly expressed. A patient-centered approach which encourages self-sufficiency and self-management and promotes external support systems is recommended.

## 1 Introduction

Inflammatory bowel disease (IBD) includes the chronic, lifelong intestinal inflammatory disorders of ulcerative colitis (UC) and Crohn's disease (CD) (1, 2). With a rising global incidence among children and adolescents (3, 4), IBD significantly impacts young adults aged 18–29 years—a critical developmental period marked by identity formation, career establishment, and social relationship development (5). The protracted and complex journey towards an IBD diagnosis, coupled with uncertain treatment outcomes, profoundly affects physical health, psychological well-being, and social functioning in this population (6, 7). These challenges are exacerbated by the complexities of disease management, limited external support, and barriers to social adaptation (8, 9).

Qualitative investigations have particularly enriched our understanding of the illness experience in this population, identified several core themes characterizing the young adult IBD experience, including uncertainties in life planning, psychological distress, relationship challenges, and identity transformation (9–12). These studies, along with others in the field, have effectively mapped the trajectory from initial diagnosis through various adaptation phases, demonstrating both the significant burdens and remarkable resilience exhibited by young adults with IBD. However, despite these valuable contributions, some gaps remain in our understanding of how young adults with IBD navigate the complex interplay between developmental tasks and illness management. Mechanisms through which social and psychological factors influence coping strategies in young patients with inflammatory bowel disease (IBD) during the transitional phase, Specifically, the dynamic interplay between intrinsic psychological resilience and external support systems in jointly fostering positive health outcomes, warrants further investigation.

Grounded in this context, the present study employs qualitative methods to examine the lived experiences of young adults with IBD, and elucidate the internal and external factors that shape their coping strategies. By doing so, it aims to identify nuanced support needs during this critical phase, thereby informing the development of targeted psychosocial and clinical interventions.

## 2 Methods

### 2.1 Participants

Purposive and snowball sampling was used to identify IBD patients aged 18–29 years presenting for outpatient or inpatient care at the Gastroenterology Department of a tertiary hospital in Jiangsu Province between September 2023 and January 2024. Eligible patients were recruited in person during their clinic visits. This included 10 patients with ulcerative colitis and 15 with Crohn's disease. Ultimately, 7 patients with ulcerative colitis and 13 patients with Crohn's disease agreed to participate in the study. The primary reasons for patients declined participation included: (1) lack of available time to complete the interview, (2) parental refusal, and (3) in one instance, the patient's active ulcerative colitis symptoms coupled with a stated reluctance to discuss defecation-related issues with individuals outside his care givers. Inclusion criteria were: (1) IBD diagnosis according to established clinical guidelines, (2) in mild to moderate disease activity or remission, disease activity in patients with Crohn's disease was assessed with the short Crohn's Disease Activity Index (CDAI) (13), whereas the Modified Mayo Endoscopic Score (MMES) (14) was used for patients with ulcerative colitis, (3) age 18–29 years, (4) ability to communicate effectively in Chinese with basic comprehension and expression skills. Exclusion criteria were: (1) severe disease leading to heart, brain, lung, kidney or other organ impairment, (2) inability to complete interviews, (3) history or current diagnosis of severe psychiatric disorder, (4) recently diagnosed patients, who disease duration <6 months. Sampling continued until data saturation when 20 participants were enrolled and were assigned codes P1–P20 to ensure anonymity (Table 1). Experiments were performed in accordance with the Declaration of Helsinki (amended 2023). Written informed consent was given by all participants for use and publication of data and ethical approval was granted by the Ethics Committee, the First Affiliated Hospital with Nanjing Medical University (approval number: 2023-SR-881).

**Table 1:** Demographic and clinical characteristics of young adults with inflammatory bowel disease (*n* = 20).

Patient ID	Gender	Age (years)	Education level	Occupation	Disease type	Disease duration (years)	Disease status	IBD-related surgery history
P1	Male	23	Bachelor's degree	Student	UC	3	Remission	No
P2	Male	19	High school	Student	CD	1	Active	No
P3	Male	21	Associate degree	Unemployed	CD	2	Remission	Yes
P4	Male	24	Bachelor's degree	Office worker	CD	2	Active	No
P5	Male	20	High school	Student	CD	2	Remission	Yes
P6	Male	18	High school	Student	CD	1	Remission	Yes
P7	Female	18	High school	Academic leave	UC	1	Active	No
P8	Female	24	Bachelor's degree	Office worker	CD	2	Remission	No
P9	Male	28	Master's degree	Teacher	UC	7	Remission	No
P10	Female	27	Bachelor's degree	Unemployed	UC	2	Active	No
P11	Female	25	Bachelor's degree	Office worker	CD	1	Active	No
P12	Male	20	High school	Student	UC	2	Remission	No
P13	Female	18	High school	Academic leave	CD	1	Active	No
P14	Female	27	Associate degree	Self-employed	UC	2	Remission	No
P15	Male	23	Bachelor's degree	Office worker	CD	4	Remission	Yes
P16	Male	20	High school	Student	CD	5	Remission	Yes
P17	Female	18	High school	Student	CD	3	Remission	No
P18	Female	26	Master's degree	Academic leave	UC	1	Active	No
P19	Male	21	High school	Student	CD	3	Remission	No
P20	Female	21	Associate degree	Office worker	CD	2	Remission	No

### 2.2 Research methods

#### 2.2.1 Rationale for a descriptive phenomenological approach

This study utilized a descriptive phenomenological framework to elucidate the essential structures of illness experience among young adults with inflammatory bowel disease. This methodological approach was deliberately chosen for its particular strength in examining subjective phenomena that remain inadequately characterized in the current literature and for its capacity to reveal fundamental meaning structures inherent to human experience (15). The phenomenological conception of the “lifeworld” as an irreducible totality aligns precisely with our investigation into the essence of transitional experiences, which inherently span biological, psychological, and social domains. Unlike methodologies focused on quantification or causal inference, phenomenology facilitates a profound engagement with subjective realities through systematic analysis of experiential accounts. Consequently, the phenomenological framework offers rigorous methodological grounding for examining how young adults with IBD perceive, interpret, and cope throughout their illness trajectories.

#### 2.2.2 Data collection

Qualitative phenomenological data were collected by semi-structured interviews conducted by two qualified researchers from the IBD center, one researcher is responsible for conducting interviews, while another is responsible for observation and documentation. Both interviewers are experienced clinical nurses with specialized training in qualitative research methodology; one of the researchers additionally holds a Master of Science in Nursing degree. To ensure consistency in data collection, both interviewers followed the same semi-structured interview guide and participated in joint training sessions. Regular debriefing sessions were held between interviewers to discuss emerging themes and maintain consistency in probing techniques. An interview plan based on research objectives and team discussions was formulated and revised following two pilot interviews with IBD patients in August 2023:

Could you share your understanding of inflammatory bowel disease (IBD)?
Could you describe your experience of becoming ill and your journey through the healthcare system?
What impacts and changes has IBD brought to your life? What thoughts and feelings do you have about these?
How have you dealt with these impacts and changes?
What support or help have you received during this process? What additional support do you feel you need?
What are your hopes and plans?

Participants were informed of the study's purpose and content, and interviews were audio-recorded and non-verbal cues documented. Interviews were conducted in a quiet, comfortable and private conference room and were of 45–60 min duration. During the interview, observe the subject's emotional and physical state to avoid influencing their subjective opinions. If there are any uncertainties regarding the interview content, promptly paraphrase the information back to the interviewee to confirm its accuracy.

Interview data collection continued until thematic saturation was reached (16). This was determined by tracking the emergence of new themes in a saturation table (17). Data collection ceased when no new themes were identified across three consecutive interviews. Saturation was first noted after the 17th interview, three subsequent confirmatory interviews yielded no new information, thus finalizing the sample size at 20 participants.

#### 2.2.3 Data analysis

Audio recordings were transcribed verbatim within 24 h of the interview and annotated with non-verbal observations. NVivo 12.0 software was used for data organization and analysis performed by Colaizzi's seven-step phenomenological method. Themes were inductively derived and refined through team discussion.

#### 2.2.4 Quality control

Trust, confidentiality and rapport were established with participants prior to interviews with times and locations agreed in advance. Participants were encouraged to express genuine experiences and feelings by repetition, probing, clarification and summarization on the part of the researcher with minimal interruption and the avoidance of leading questions. Observations regarding tone, facial expressions, emotional shifts and body language were recorded. Viewpoints were confirmed with the patient contemporaneously to ensure accuracy. Two researchers performed independent and iterative analyses of transcripts to compare coding and thematic interpretations and resolve discrepancies. Key findings were confirmed by reference to participants.

#### 2.2.5 Reflexivity and positionality management

As clinician-researchers affiliated with the IBD Center, we acknowledged how our dual roles potentially influenced multiple research phases: established trust relationships may have increased participation rates; pre-existing rapport possibly facilitated more open disclosure during interviews; and clinical backgrounds inherently shaped analytical perspectives. To mitigate these influences, we implemented several strategies including: bracketing of pre-existing assumptions during analysis, participant reassurance of confidentiality separate from clinical care, and peer debriefing with non-clinical researchers.

## 3 Results

### 3.1 Theme 1: the experience of complex negative emotions

#### 3.1.1 Disrupted daily life

Recurrent abdominal pain, diarrhea, persistent fatigue and malnutrition caused noteworthy disruption to daily routines with impacts on diet and exercise. Frequent medical visits impinged on academic/work schedules. Participants felt that IBD had shattered their previous lives:

P5: “*Since my second year of high school, I've constantly had stomachaches and diarrhea. I can't eat this or that, and I have to rush to the bathroom right after class. It's really made me feel emotionally down.*”
P18: “*I've been to hospitals in Beijing and Shanghai… This year, I've either been seeing doctors or on the way to see one. I've had to take a leave of absence from school now… I keep passing blood. The doctor said I have severe anemia and low protein, which is why I always feel exhausted and light-headed when walking.*”
P10: “*It affects normal life. During my last severe flare-up, I couldn't work for a while and had to quit my job. So, the financial burden is heavy too.*”

#### 3.1.2 Restricted self-development

IBD derailed academic and career plans, leading to feelings of restricted self-exploration and growth:

P12: “*The doctor said UC will relapse if I'm not careful, and I need long-term medication. I originally wanted to go to military academy, but I had to give that up. It's frustrating.*”
P16: “*It affected my career planning. This disease means I can't handle stress or overwork; pressure makes my stomach hurt and gives me diarrhea… I'm studying computer science now, but I don't want to work in IT later—it's too demanding; I couldn't handle it.*”

#### 3.1.3 Uncertainty and worry about the future

The progressive nature of IBD and unpredictable treatment outcomes caused significant worry about future academic/career paths and intimate relationships, such as marriage:

P4: “*I've heard that with Crohn's disease; you'll inevitably need bowel surgery at least once. It'll probably be my turn eventually too.*” (Frowning, silently looking downwards.)
P20: “*Since getting sick, I take frequent sick leave, and my work performance is unstable. I don't know if the biologics will stabilize my condition, but at least I hope it won't affect my job.*”
P8: “*I know a fellow patient whose son also has Crohn's. This disease affects quality of life and might be passed on to the next generation. My parents keep urging me to get married, but I'm unsure if I should even date or get married.*”

#### 3.1.4 Conflict between the desire for independence and reality of dependence

Young adults with IBD expressed a strong desire for independence, typical of their age, but were forced to rely on parents or caregivers for disease management. The result was profound internal conflict:

P3: “*Because of my illness, I haven't found a job yet. My parents give me money. Honestly, I don't like this; I'm an adult, yet they still manage everything I do. But I have no choice; I still need their help with wound care.*” (Speaker had received perianal abscess surgery).
P17: “*Since I got sick, my mom has taken care of all my daily needs and accompanies me to appointments. I know I should be more independent, but I still can't manage without her right now.*”

### 3.2 Theme 2: disease coping strategies

#### 3.2.1 Active coping

Reliable family support and greater self-reliance allowed some patients to manage their IBD. Others were able to adapt to the disease state by adjusting their perceptions of illness and with the help of peer support. Several participants described experiences that align with the conceptual framework of post-traumatic growth. For example, in confronting illness-related challenges, they recognized a previously untapped personal strength through sustained engagement in treatment and self-management. This enhanced capacity to cope not only helped them endure but also fostered personal development that transcended their pre-illness sense of self:

P6: “*My parents bought commercial insurance for me, so the medication costs aren't a huge burden for the family. With their support and encouragement, I've been actively cooperating with treatment.*”
P1: “*I treat this disease like high blood pressure—taking my medication on time every day. I also pay more attention to my diet.*”
P9: “*I joined an IBD support group. Encouraged by other patients, I stick to exercising, am very careful with my diet, and avoid staying up late. Now I feel physically better than before I got sick and haven't had a flare-up for two or three years.*”
P19: “*I did exclusive enteral nutrition for a while—only nutritional formula, nothing else to eat. It was tough, you know? But I persevered. That experience made me realize I*”*m stronger than I thought; I can handle various challenges.*”

#### 3.2.2 Passive avoidance

Some participants adopted avoidant or skeptical coping strategies, often rooted in limited confidence in treatment efficacy or discomfort regarding symptoms such as diarrhea and changes in body image. These responses typically manifested as social withdrawal or expressed skepticism toward disease management. Furthermore, avoidant coping among young adults with IBD demonstrated contextual and phase-specific patterns, with a pronounced tendency toward such strategies during disease flares or critical treatment transitions—such as in the pre-surgical phase:

P7: “*The chief physician gave me a book on self-management, saying long-term management is key for this disease. But I've tried several medications with poor results. Surgery seems inevitable eventually, so what's the point of talking about management?*”
P13: “*Hmm… (silence) After getting sick, I lost thirty pounds, and now I have to eat through a tube (Nasoenteric tube)… After taking leave from school, I stopped contacting my old classmates. I don't want them to see me like this.*”
P5: “*Going out means constantly looking for a bathroom. It's awkward to explain why, so I basically never go out with anyone except family.*”

### 3.3 Theme 3: external support needs

#### 3.3.1 Family support

Participants were more likely to engage with active coping if they received material support, daily care and emotional support from family members. Participants expressed a need for family understanding, support and practical help:

P15: “*After I was diagnosed with Crohn's following bowel perforation surgery, my mom bought recipe books specifically to cook for me. My dad often took me out to relax and encouraged me to seek treatment actively. They helped me through that dark time.*”
P18: “*My peers are working and earning money, but I had to take a leave of absence from school because of my illness. I hope they [the participant's parents] can understand my inner struggle. Without their support, I might not have been able to persevere.*”

#### 3.3.2 Professional support

Information and health guidance from healthcare professionals (HCPs) allowed patients to evaluate treatment options and make informed medical decisions:

P7: “*The doctor said for my condition, I either need surgery to remove the damaged bowel or long-term medication. I don't understand which is better; I really need guidance from your medical staff.*”
P9: “*I feel Dr. Zhang and the head nurse have been incredibly helpful. When I was first diagnosed, I knew nothing. Hearing I needed steroids made me very resistant. It was their patient explanation of the treatment plan, assuring me steroids weren't long-term, that convinced me to take the medication.*”

#### 3.3.3 Social support

Support from peers, schools and patient organizations alleviated feelings of isolation and helplessness and promoted self-reliance:

P8: “*I often participate in IBD charity events. Talking with other patients and seeing those who've lived with the disease for over ten years looking healthy like anyone else gives me confidence that I can do it too.*”
P12: “*After my first hospitalization, when I went back to school, my teacher specifically came to talk to me and comfort me. My classmates also came to my home to help me catch up on schoolwork. It made me feel less alone, and catching up didn't seem so difficult anymore.*”

## 4 Discussion

### 4.1 Navigating developmental disruptions: IBD's impact on transition to adulthood

The present study reveals that inflammatory bowel disease significantly disrupts the core developmental tasks that define the transition to adulthood. Young adults in our cohort consistently reported that IBD interfered with self-identity exploration, educational attainment, career advancement, and the formation of intimate relationships. This perceived disruption of their expected life trajectory generated substantial psychological distress, characterized by uncertainty about the future and resignation from previously held aspirations. Many participants described their future plans in terms of “*don't know*” or “*uncertain*” with some abandoning long-term goals entirely, creating a pronounced gap between pre-illness aspirations and current realities. Concerns about disease inheritance led some to hesitate about pursuing intimate relationships. Young adults also experienced embarrassment caused by diarrhea and pain which impeded social interactions and led to withdrawal and isolation. These findings align with Qualter et al. (18), who similarly documented the profound psychosocial consequences of IBD during young adulthood. This study found that impaired autonomous treatment decision-making, disease management or lack of stable income made many young adults dependent on parents or other caregivers despite their strong desire for independence. This highlights a significant tension for young adult: a particularly salient conflict between the developmental drive for independence and the practical dependencies created by IBD.

The active participation in and assumption of responsibility for disease management is crucial for IBD patients transitioning from pediatric to adult care (19). Our findings suggest that effective support should simultaneously address both medical and developmental needs. This can be achieved through balanced strategies that promote patient autonomy while meeting transitional care requirements—a key consideration for healthcare providers. Such strategies may involve facilitating opportunities for young adults to gradually assume self-care responsibility while ensuring appropriate support from parents and multidisciplinary team.

### 4.2 Coping strategies of young adults with IBD

This study identified distinct coping strategies among young adults with IBD, shaped by their disease experiences and self-perception. Participants who demonstrated robust emotional regulation and maintained confidence in their treatment were more likely to adopt positive coping strategies. These individuals tended to view IBD as a manageable chronic condition which promoted treatment engagement and adherence.

By contrast, patients who had experienced unsatisfactory treatment outcomes or suffered from low confidence tended to adopt passive avoidant coping strategies. This divergence in coping strategies aligns with existing evidence on coping adaptation in chronic illness (7). The association between coping strategies and psychosocial outcomes appears mediated through cognitive-emotional pathways. Passive avoidance may lead to more severe psychological distress in IBD patients whereas active coping may improve mental health and enhance quality of life (20). Thus, actions that foster cognitive shifts also serve to build resilience and facilitate growth beyond their pre-illness state (21).

The experiences of participants who successfully coped suggest that enhancing self-reliance is a pivotal factor associated with active coping, positioning it as a potential critical focus for interventions aimed at preventing avoidance. Education to promote IBD self-management and personalized psychological support has been shown to help patients to reframe illness perception, discover internal resources and strengthen confidence in disease management (22). In addition, IBD charities and peer support were found to encourage positive coping strategies among the current cohort of young adults. Participants' also narratives implied a potential role for clinical healthcare providers in health education, psychological support, and facilitation of peer connections, as part of a comprehensive support system.

This study reveals that coping in young adults with IBD follows contextual and phase-specific patterns, aligning with the findings of Gelech et al. (6). Notably, the use of avoidance coping is heightened during acute disease flares or critical treatment transitions. Consequently, understanding these behaviors necessitates moving beyond reductive classifications of avoidance as uniformly maladaptive. Clinical interventions need distinguish between short-term adaptive withdrawal and entrenched avoidance, and between healthy skepticism and generalized disengagement. The goal is to offer contextualized—not merely categorical—support. Ultimately, a balanced approach that acknowledges the protective role of temporary avoidance, while actively cultivating proactive coping strategies, may more fully honor the complexity of young patients' illness experiences in transition.

### 4.3 The role of social support systems in enhancing coping capabilities

Our study found a multi-layered social support ecosystem that significantly influences coping outcomes among young adults with IBD. Participants consistently highlighted four primary support sources: familial relationships, healthcare providers, peer networks, and institutional accommodations from educational/workplace environments.

The centrality of social support aligns with the 2023 priority setting exercise that identified “How to get support to cope with IBD” as a key concern for this population (23). Specifically, patients reporting well-structured support systems demonstrated enhanced treatment adherence and psychological adaptation. Family support has previously been shown to improve treatment adherence, encourage good disease management and promote a healthy psychological state (24, 25). Family health education may be given by HCPs to enhance disease care and emotional support while guiding the young adult towards self-reliance by promoting treatment decision-making and alleviating negative emotions (26, 27). Nurses are appropriate providers of informational support and long-term follow-up, including disease management programs and case management, which may enable patients to cope. Support from peers, patient organizations and schools was found too to mitigate feelings of isolation and helplessness among the current cohort. Thus, integrating structured peer support with school- and community-based adaptations into transitional care emerges as a promising approach. Collectively, the findings affirm the value of a coordinated support system involving multidisciplinary collaboration (28). Ultimately, strengthening these interconnected support layers, as evidenced by the participants who thrived, appears to create sustainable foundations for long-term adaptation.

## 5 Limitations

This study has several methodological limitations that warrant consideration. The monocentric design at a tertiary hospital in Jiangsu Province may affect the transferability of findings to primary care settings or regions with different healthcare resources. While purposive and snowball sampling enabled rich qualitative data collection, However, the perspectives of patients in the severe disease activity phase may not be fully represented, as these patients were excluded from the study due to unstable conditions or excessive symptom burden. The cross-sectional interview design further limits observation of how coping strategies evolve throughout disease trajectories. The cultural context of the study sample may influence generalizability, as observed emphasis on family support and hierarchical patient-provider relationships reflects distinctive aspects of the Chinese healthcare system. Additionally, while peer debriefing was employed, the clinician-researcher dynamic may have introduced some social desirability bias in participant responses.

## Data Availability

The original contributions presented in the study are included in the article, further inquiries can be directed to the corresponding author.
